# A Case for Diagnosis

**Published:** 1908-04-04

**Authors:** 


### Medicine.

### A CASE FOR DIAGNOSIS.

A male child aged two years was admitted to a hospital, having been ill for a month with vomiting (chiefly after food), feverishness, pains in the head. and sleeplessness. It was extremely fretful, screaming when touched. There was a history of phthisis in the father's family, and the child itself, when fourteen months old, had suffered from empyema, for which resection of a rib had been performed. Complete recovery followed, and no other illness had occurred until the present one began. There was no history of injury.

On admission, the temperature in the axilla was 103.6° F. There was no special intolerance of light, nor retraction of the head. *Tâche cérébrale* was well marked. No evidence of disease was found on examining the chest or abdomen, and the child was in general well-grown and healthy-looking. The pulse was regular and the respirations were quiet. Next day the temperature fell to 98° F., and the child appeared to be quite well.

During the next two months, every other day, or occasionally at intervals of two or three days, the symptoms recurred, namely: a rise of temperature to 103° F. or 104° F., falling to 97° F. or 98° F. within twenty-four hours, accompanied by vomiting, extreme irritability, screaming when touched, pain in the head, and flushed cheeks. Between the attacks the child was placid, amiable, and apparently quite well, taking its food regularly and sleeping naturally. The pulse was always regular, and no signs of disease were discoverable in the heart, lungs, or abdomen. The optic discs were often examined, but appeared normal. There was no ear discharge, and the drums looked healthy. The urine was normal, the bowels were regular; blood-examinations showed no abnormality. Various drugs, including iodides, mercury, and quinine, were given, but without apparent effect.

Nine weeks after admission the child began to suffer from a slight cough, and a few scattered rhonchi were detected. The attacks still continued, and the patient began progressively to lose flesh. In other respects the child continued about the same, and he was described as very intelligent and as making progress in talking. This state continued for another six weeks, when, one day, after the usual rise of temperature, the boy became very drowsy and difficult to arouse. If he was put into the sitting posture there was a tendency to fall to the left side. This condition recurred several times, but in the intervals he became quite conscious and sensible.

Ten days after the first onset of drowsiness convulsions occurred for the first time, especially on the left side. The optic discs were now seen to be slightly inflamed, and this rapidly increased. The pulse also began to be irregular, and a week after the convulsions appeared—that is to say, more than six months after the illness began—the child died comatose. No abnormal symptoms other than those recorded were observed.

### SOLUTION.

The condition was clearly a very difficult one to explain satisfactorily. At first, some form of acute meningitis was suspected, but the length of survival excluded all but the posterior basal form, and there was none of the severe retraction of the head, and so forth, usually associated with that at one stage or another. Cerebral tumour and cerebral abscess each suggested themselves later, and of the two abscess seemed rather the more likely, on account of the previous history of empyema and because of the pyrexial attacks.

There was nothing to assist in localising either abscess or tumour, and difficult though the case is of diagnosis, at least one thing is taught by it, namely, not to advise trephining unless there are good grounds for it. The post-mortem findings showed that nothing could have been gained by trephining, and that it was not impossible for the lesions to have got well by themselves. It is much more difficult sometimes to hold one's hand rather than "do something."

The following appearances were found:—The head was of normal size. There was no sign of rickets. The skull-cap was normal in regard to development and ossification. The membranes and the sinuses of the brain were normal, and so were the ears. The convolutions were a little flattened by moderate distension of the ventricles with clear cerebro-spinal fluid. The walls of the ventricles were thinner than usual, the grey matter of the cortex being relatively less atrophied than the white matter. The veins of Galen were well marked and not thrombosed. Everywhere the cerebral substance was firm. The brain weighed 41 ounces before the fluid within it escaped.

Immediately behind the optic chiasma there was a small flat patch of cream-coloured deposit about half an inch in diameter. Similar patches of minute size were situated in both Sylvian fissures, and on the centre of the inferior surface of both halves of the cerebellum. The deposits were apparently of inflammatory origin, but not tuberculous. It is conceivable that they were local areas of softening left behind after the greater part of the exudate of a posterior basal meningitis had already disappeared, in which case it was sheer ill-fortune that the patient did not recover entirely. Moreover, if this were so, the case would illustrate the way in which posterior basal meningitis may cause hydrocephalus, the primary origin of which is now held to be a cured posterior basal meningitis in the majority of cases.

The only other sign of disease found in the body was the universal adhesion of the left pleural surfaces, where the empyema had formerly been. There were no enlarged nor caseous lymphatic glands in any part of the body.

